# Synthesis and Characterization of Cellulose Nanofibril-Reinforced Polyurethane Foam

**DOI:** 10.3390/polym9110597

**Published:** 2017-11-10

**Authors:** Weiqi Leng, Jinghao Li, Zhiyong Cai

**Affiliations:** 1 U.S. Department of Agriculture, Forest Service, Forest Products Laboratory, Madison, WI 53726, USA; wleng@fs.fed.us; 2 Department of Biomaterials, International Center for Bamboo and Rattan, Beijing 10000, China

**Keywords:** cellulose nanofibrils, polyurethane foam, reinforced nanocomposite

## Abstract

In this study, traditional polyol was partially replaced with green, environmentally friendly cellulose nanofibrils (CNF). The effects of CNF on the performance of CNF-reinforced polyurethane foam nanocomposites were investigated using scanning electron microscopy, Fourier transform infrared spectroscopy (FT-IR), X-ray diffraction (XRD) analysis, thermogravimetric analysis (TGA), differential scanning calorimetry (DSC), dynamic mechanical analysis (DMA), and a compression test. The results showed that the introduction of CNF into the polyurethane matrix not only created stronger urethane bonding between the hydroxyl groups in the cellulose chain and isocyanate groups in polymethylene polyphenylisocyanate, but also developed an additional filler–matrix interaction between CNF and polyurethane. With the increase of the CNF replacement ratio, a higher glass transition temperature was obtained, and a higher amount of char residue was generated. In addition, an increase of up to 18-fold in compressive strength was achieved for CNF-PUF (polyurethane foam) nanocomposites with a 40% CNF replacement ratio. CNF has proved to be a promising substitute for traditional polyols in the preparation of polyurethane foams. This study provides an interesting method to synthesize highly green bio-oriented polyurethane foams.

## 1. Introduction

Polyurethane foam, first produced and then commercialized in the 1950s, have attracted much attention due to their low density, high mechanical properties, and use in a wide variety of applications including the construction and automotive industries, depending on the type of foam [1,2,3]. PUF has been used extensively since their commercialization. The global PUF market value was about 49 billion US dollars in 2015, and there is expected growth up to 92 billion US dollars by 2024 [4]. PU foams are usually prepared by the reaction of petroleum-based polyols (either polyol polyether or polyol polyester) with isocyanate, forming urethane linkages. In addition, catalysts, surfactant and blowing agents are needed to regulate their properties and cell morphology [5,6]. There are wide varieties of polyols and poly-isocyanates that can be used to synthesize PUF. The foam properties vary significantly depending on the selected raw materials. The PUF can be flexible, semi-rigid, and rigid [5].

Although PUF has many merits, there is a significant drawback that causes much environmental concern. The raw materials for PUF are petroleum-based and non-renewable, which are difficult to degrade in nature [6]. There is an urgent need to find raw materials that are environmentally friendly and competitive with petroleum-based counterparts, in terms of price and properties [7,8]. Extensive research has been conducted to modify the polyols and make them biodegradable [9]. However, the chemically modified PUF had inferior mechanical properties compared to its petroleum-based counterpart [10]. Hence, additives were introduced to improve the mechanical properties of PUF. The idea was that natural materials containing hydroxyl groups could play the same role as polyols did [11]. Many natural materials including starch, soy flour and cellulose were extensively investigated, with focus on cellulose and its derivatives, due to their extraordinary properties [6].

Cellulose is the most abundant natural polymer in the world with global reserves of up to 75 billion tons, which accounts for approximately 40% of plant biomass [12,13,14,15,16,17]. Cellulose can be obtained from a variety of sources including wood, non-woody plants, agricultural residues, algae and bacteria [18]. Cellulose consists of several hundred to over ten thousand β-1,4-d-linked glucose chains, in which the glucose units are joined by single oxygen atoms (acetal linkages) between the C-1 of one glucose unit and the C-4 of the next unit. There are a large number of hydroxyl groups on the glucose unit that can easily form hydrogen bonds with each other to hold the chain together [19,20]. Cellulose I and II are the first and second most extensively studied allomorphs. Cellulose I has the native crystalline structure, and is insoluble in water. It can be converted to cellulose II, with a crystalline structure via a modification or regeneration treatment, during which the native crystalline structure is altered, and the mechanical properties decrease [12,21,22,23,24].

Cellulose nanofibrils (CNFs), an aggregation of 10–50 cellulose elementary fibrils with a complex web-like network structure, have gained increasing interest due to their excellent properties including high Young’s modulus (estimated at ~140 GPa in the crystal region along the longitudinal direction) and specific strength, making it an ideal building block for products with desirable mechanical properties [25,26]. CNFs have a diameter in the range of 5 to 50 nm and length of several μm, respectively [27]. They also exhibit a hierarchical order in the supramolecular structure and organization, high aspect ratio, high surface area, and reactive surfaces containing –OH groups. All these unique characteristics make CNF a promising candidate as a reinforcing material and may at least partially replace polyols [28,29,30,31,32,33,34,35].

CNF is usually manufactured from wood pulp via mechanical defibrillation with chemical, enzymatic or physical pre-treatments. Various methods have been employed to defibrillate cellulose, e.g., blending, high-pressure homogenization, steam explosion, and grinding [14,36,37,38]. However, all means of mechanical disintegration of cellulose require high energy inputs. Consequently, chemical pretreatment methods have been developed to reduce energy use, including 2,2,6,6-tetramethylpiperidine-1-oxyl radical (TEMPO)-mediated oxidation, carboxymethylaion, periodate-chlorite oxidation, enzymatic reactions, acidified chlorite, ultra-sonication, or a combination of two or more of these methods [27,39,40,41,42,43].

CNF is usually dispersed in water and stored in a cold room. However, in PUF synthesis, water is a popular blowing agent. Special forms of CNF should be used to replace traditional polyols in the production of PUF. Research has been conducted using up to 10% CNF as the additive to improve the mechanical properties of PUF. However, higher CNF weight ratios have rarely been investigated [44,45,46]. In this study, a CNF weight ratio (based on total weight) of up to 20% was added to replace petroleum based polyols, i.e., polyethylene glycol (PEG-400). The compressive properties were significantly improved. In addition, the morphology, thermal stability, and spectroscopic characterization of the foam composites were analyzed in this study.

## 2. Materials and Methods

### 2.1. Materials

Polymethylene polyphenylisocyanate (PAPI^TM^ 27 Polymeric MDI) was donated by Dow Chemical, Midland, MI, USA, Polyethylene Glycol (PEG-400) was procured from Sigma Aldrich, St. Louis, MO, USA. Spray-dried CNF was supplied by the process development center at University of Maine. DABCO T12 catalyst and DABCO DC5357 surfactant were donated by Air Products, Allentown, PA, USA. Deionized water was used as the blowing agent.

### 2.2. Fabrication of Pure PUF and CNF-PUF

A detailed foaming formulation is listed in Table 1. For the PUF control, PEG-400, DABCO T12, DABCO DC5357, and deionized water were first mixed for about 5 min under mechanical stirring in a plastic beaker until homogeneous mixture was obtained. For the CNF-PUF, a specific amount of spray-dried CNF was first added to PEG-400 and mixed for 5 min under mechanical stirring in a plastic beaker until a homogeneous mixture was obtained. DABCO T12, DABCO DC5357, and deionized water were then added into the mixture and further stirred for another 5 min. Finally, PAPI 27 was added into the mixture and vigorously stirred for 30 s, after which the foaming process started. The final products were cured overnight in a vacuum oven at 50 °C The cured foams were then cut into different sizes and conditioned at 20 °C and 50% relative humidity conditioning room before characterization. In this study, the –NCO/–OH index was set at 1.1 to ensure complete reaction of all –OH groups.

### 2.3. Characterization

#### 2.3.1. Scanning Electron Microscopy (SEM)

A scanning electron microscope (Zeiss LEO 1530 Gemini, Oberkochen, Germany) with an acceleration voltage of 5 kV was used to evaluate the morphology of PUF and CNF-PUF samples. Thin slices of 10 mm × 10 mm × 3 mm were cut from the foams using a stainless steel blade and then mounted onto carbon tapes on the aluminum stubs. Samples were then sputter coated with gold (Denton High Vacuum Coating System, Moorestown, NJ, USA) for 1 min under vacuum. The working distance was set at 5 mm.

#### 2.3.2. Fourier Transform Infrared Spectroscopy (FT-IR)

Both pure PUF and CNF-PUF samples were characterized by a Thermo Nicolet iZ10 FTIR spectrometer, attenuated total reflection (ATR) probe (Thermo Scientific, Verona, WI, USA) using a smart iTR^TM^ Basic accessory. A diamond crystal with 45° incident angle was used. The absorbance spectra were taken for an average of 64 scans in the range of 4000–400 cm^−1^ with resolution of 4 cm^−1^. The spectra were baseline corrected, averaged, and normalized using Omnic v9.0 software (Thermo Scientific, Verona, WI, USA).

#### 2.3.3. X-ray Diffraction (XRD)

XRD patterns of PUF and CNF-PUF samples were obtained with a Bruker Discover 8 diffractometer (The Woodlands, TX, USA) using a Cu Kα rotation tube at 50 kV and 1000 μA with a scanning range from 5° to 50°. The scanning speed was 10°/min. The crystallinity index (CI) was expressed by measuring the peak height of the crystalline area (*I*_002_) and amorphous area (*I_am_*), as shown in Equation (1) [47,48]:

(1) $$\mathrm{CI}\left(\%\right)=\frac{I_{002}-I_{am}}{I_{002}}*100\%$$

#### 2.3.4. Thermogravimetric Analysis (TGA) and Differential Scanning Calorimetry (DSC)

The foam’s thermal stability was tested using a Pyris 1 TGA (PerkinElmer, Shelton, CT, Waltham, MA, USA). Samples were heated from 25 to 800 °C at a heating rate of 5 °C/min under nitrogen gas environment with a flow rate of 20 mL/min. Approximately 5 mg of samples were used for each test. The loss of weight was recorded and normalized against the initial weight. Another group of samples were analyzed with a DSC Q2000 (TA Instruments, New Castle, DE, USA). Samples of 4–6 mg were placed in hermetic aluminum sample pans and first cooled down from room temperature to −30 °C and then heated to 400 °C at a rate of 5 °C/min.

#### 2.3.5. Dynamic Mechanical Analysis (DMA)

The viscoelastic properties of polyurethane and its nanocomposites were measured using DMA (DMA-Q800, TA instruments, New Castle, DE, USA) in penetration mode. The square samples (10 mm × 10 mm × 4 mm) were placed onto a compression clamp, and heated from 30 to 150 °C at a rate of 2 °C/ min with a dynamic strain of 0.1% and a preload force of 0.01 N at a single frequency of 1 Hz.

### 2.4. Compression Test

The compressive properties of the foam samples were tested using a universal testing machine (Instron 5544, Norwood, MA, USA) equipped with a 1 kN loading cell. The speed of the crosshead was set at 1.27 mm/min. The dimension of the samples was 12.7 mm × 12.7 mm × 12.7 mm. The compression direction was parallel to the foam rising direction. The compressive strength was calculated at 30% compression ratio. Fifteen samples were tested for each group.

## 3. Results and Discussions

### 3.1. Foam Structure

**Figure 1:**
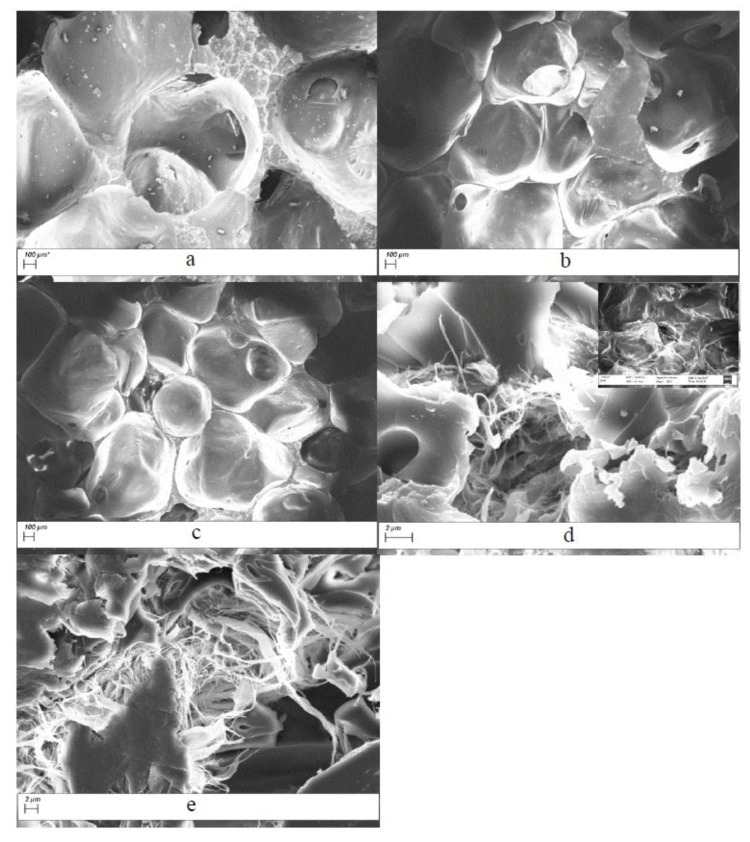
SEM images of (**a**) PUF control and CNF-PUF with (**b**) 10%, (**c**) 20%, (**d**) 30%, (**e**) 40% CNF replacement ratios.

Figure 1 shows the microstructure of the PUF control and CNF-PUFs. SEM images confirmed open-cell structure of the foams. Figure 1a shows that the microstructure of the PUF control was a simple alignment of open cells. With the introduction of CNF, the open-cell structure was disrupted, and as the CNF replacement ratio increased, there were large amounts of CNF deposited on and dispersed among the open cells, providing a chance to interact with the PUF matrix. The CNF played an important role as fillers interacting with the PUF cells, which potentially resulted in improved mechanical properties. It was reported in another study that the CNF can strongly interact through hydrogen bonding and improve the nanocomposite behavior [49].

### 3.2. Fourier Transform Infrared Spectroscopy (FT-IR)

**Figure 2:**
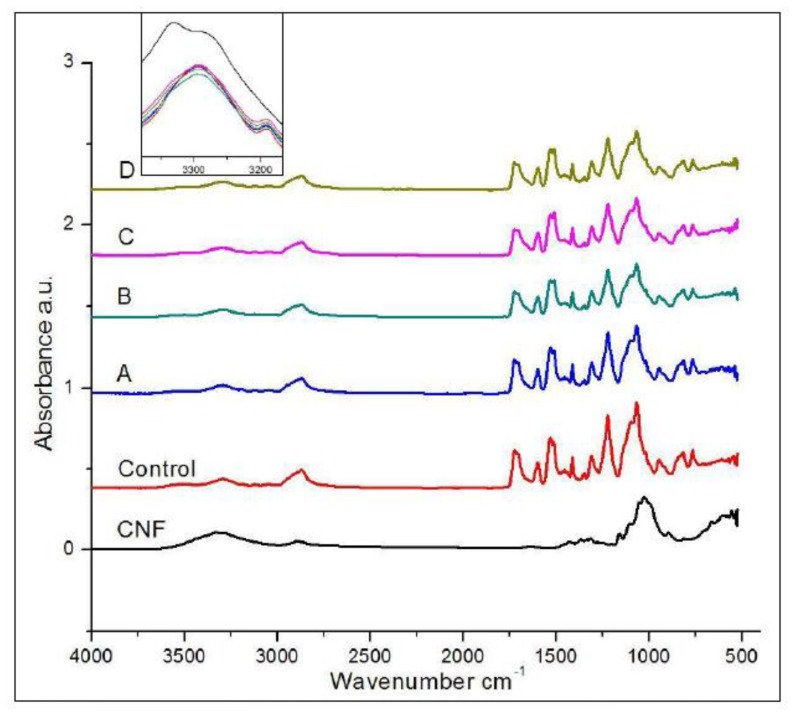
FTIR spectra of CNF, PUF control, and CNF-PUF with 10–40% CNF replacement ratios.

The FTIR spectra of CNF, PUF control, and CNF-PUF with different replacement ratios are shown in Figure 2. The –OH groups in CNF are obvious at 3300 cm^−1^, while the –OH peak shifted slightly to the right in the PUF control and CNF-PUF samples (insert in Figure 2), and the intensity dropped down sharply, compared with that in CNF. This was direct evidence showing that the –OH groups in CNF reacted with isocyanate groups in PAPI27. However, not all the –OH groups in CNF reacted with isocyanate groups; even though the isocyanate groups were overdosed in the original formulation (–OH/NCO = 1:1.1), it was possible that due to molecular Steric hindrance, some –OH groups were not accessible by the isocyanate groups. Both PUF control and CNF-PUF show peaks at 3200 and 1720 cm^−1^, which corresponded to urethane –NH stretching, and urethane carbonyl groups, respectively [50]. It was unexpected that no isocyanate peak was observed at 2270 cm^−1^, since isocyanate groups were overdosed in the original formulation. One possible reason was that excess isocyanate reacted with moisture in the air during the final cure step; since isocyanate is very reactive with –OH groups, it could capture –OH groups in the moisture and form amine and carbon dioxide [51]. The two peaks at 2850 cm^−1^ and 900 cm^−1^ were ascribed to –CH stretching and –CH bending vibrations [52]. A sharp C–O–C peak appeared at 1100 cm^−1^, which corresponded to the polyether (PEG-400) used as the polyol (not polyester) in this study [52]. In general, there was no significant difference between the PUF control and CNF-PUF in terms of functional groups, since CNF did not introduce any new functional groups into the polyurethane.

### 3.3. X-ray Diffraction (XRD) Analysis

**Figure 3:**
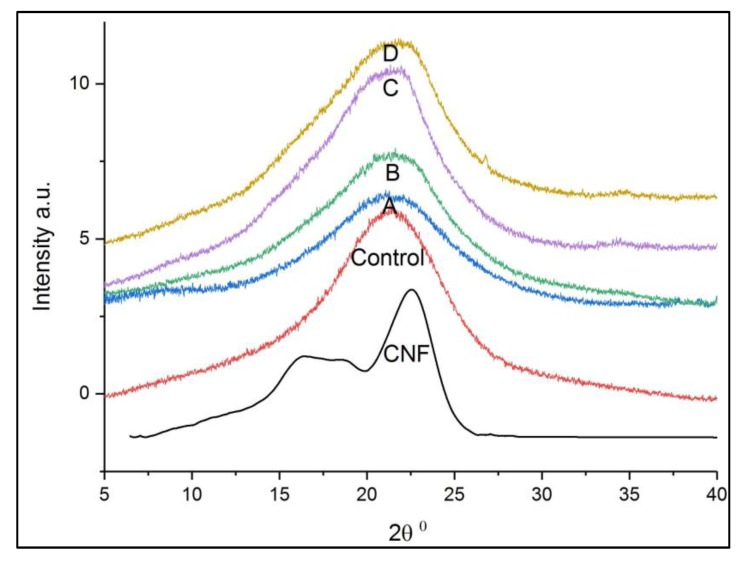
XRD spectra of CNF, PUF control, and CNF-PUF with 10–40% CNF replacement ratios.

Wide angle XRD was used to determine the effect of CNF on the macro- and microstructure changes of PUF. Figure 3 shows the XRD patterns of pure CNF, PUF control, and CNF-PUF from 2θ = 5–40°, because all the characteristic peaks were in this range. CNF had three peaks at 2θ = 16.3°, 18°, and 22.4°, corresponding to the diffractions of amorphous cellulose II, amorphous cellulose I, and crystalline cellulose I respectively [53,54]. The crystallinity of CNF was 41.7%. For the PUF control, a wide diffraction from 15–25° with a maximum peak appeared at approximately 21° [51]. It was reported that there were a few sharp peaks between 15–25°, making it easier to accurately calculate the crystallinity index [55,56,57]. All CNF-PUF composites showed similar diffraction patterns to the PUF control in Figure 3. Introducing CNF into the PUF matrix resulted in the two characteristic diffraction peaks of CNF overlapping with the PUF peak in the nanocomposites, and decreased intensity after the initial incorporation of CNF, followed by greater intensity with the increase of the CNF replacement ratio. At first, the intensity decreased because the introduction of CNF disrupted the originally uniform PUF structure and made the nanocomposite more amorphous [51,58]. Increasing the replacement ratio of CNF gradually made it more compatible with the PUF matrix and formed more uniform composites. Hence, the intensity increased again.

### 3.4. Thermal Properties of PUR and CNF-PUR

**Figure 4:**
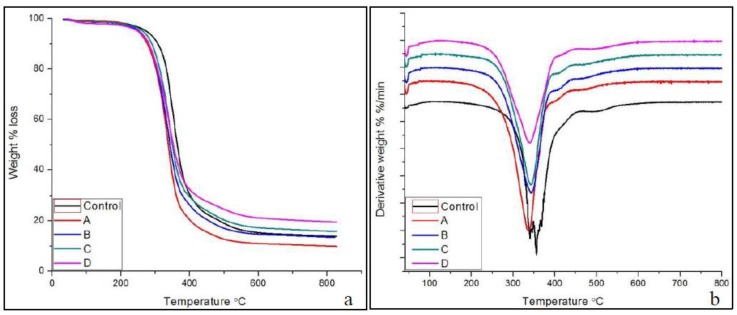
(**a**) TGA and (**b**) DTG of PUF control and CNF-PUF with 10–40% CNF replacement ratios.

Thermal stability is an important characteristic for structural materials, and polyol plays a vital role in determining thermal stability [59]. The thermogravimetry (TG) and differential thermogravimetry (DTG) (derivative weight change) results for PUF control and CNF-PUF with various CNF replacement ratios are shown in Figure 4. The TG curve (Figure 4a) shows that all CNF-PUF started to degrade at 240 °C, which was lower than the PUF control (at 270 °C). It was reported that the thermal degradation of pure CNF aerogel occurred at a temperature around 215 °C in nitrogen atmosphere, which was lower than that of PUF [60]. Hence, the initial thermal degradation temperature for CNF-PUF was lower than the pure PUF control. In addition, the DTG curve (Figure 4b) shows that the greatest weight loss occurred at around 360 °C for the PUF control and 340 °C for CNF-PUF. The incorporation of CNF also lowered the temperature for the greatest weight loss. For both the PUF control and CNF-PUF, there was only one major weight loss between 240 and 650 °C.

**Figure 5:**
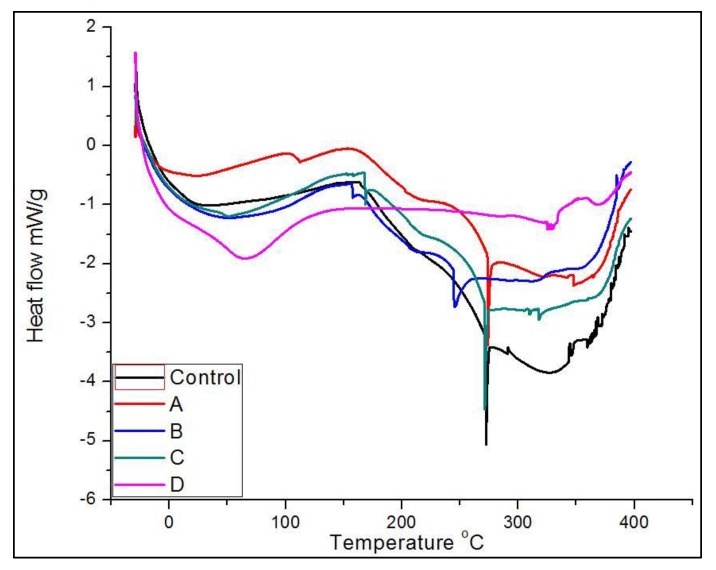
DSC curves of PUF control and CNF-PUF with 10–40% CNF replacement ratios.

The amount of char residue for the PUF control was 14% at 650 °C. For the CNF-PUF, the amount of char residue increased concurrently with the CNF replacement ratio, up to 20% gain for samples with 40% CNF replacement ratio. CNF is known as a radical scavenger during thermal degradation, resulting in higher char residue [61]. DSC analysis was further used to characterize the thermal properties of foam samples. The DSC curve (Figure 5) shows that there were three significant endothermic peaks for both PUF control and CNF-PUF. The first endothermic peak appeared at 30 °C for PUF control, and that shifted up to 70 °C for CNF-PUF with a 40% CNF replacement ratio. This first endothermic peak was related to the glass–rubber transition of the foams. The reaction between CNF and the isocyanate group resulted in a stronger cross-linking matrix than the pure PUF [49], since there are three available –OH groups in CNF skeleton. Hence, more energy was required to mobilize the foam structure, and the glass transition temperature (*T*_g_) increased after partially replacing the PEG-400 with CNF. The second endothermic peak appeared at 270 °C for both the PUF control and CNF-PUF with a CNF replacement ratio of 10%, 30%, and 40%, while that for CNF-PUF, with a CNF replacement ratio of 20%, appeared at 250 °C. The second endothermic peak was caused by the decomposition of the urea bond which was formed by the reaction of isocyanate with water, as well as to the decomposition of urethane group [45,59]. The DSC results showed that the replacement of CNF did not affect the decomposition of the urea bond. The last endothermic peak was ascribed to the decomposition of isocyanurate bonds, which occurred at temperatures above 300 °C [59].

### 3.5. Dynamic Mechanical Analysis (DMA)

**Figure 6:**
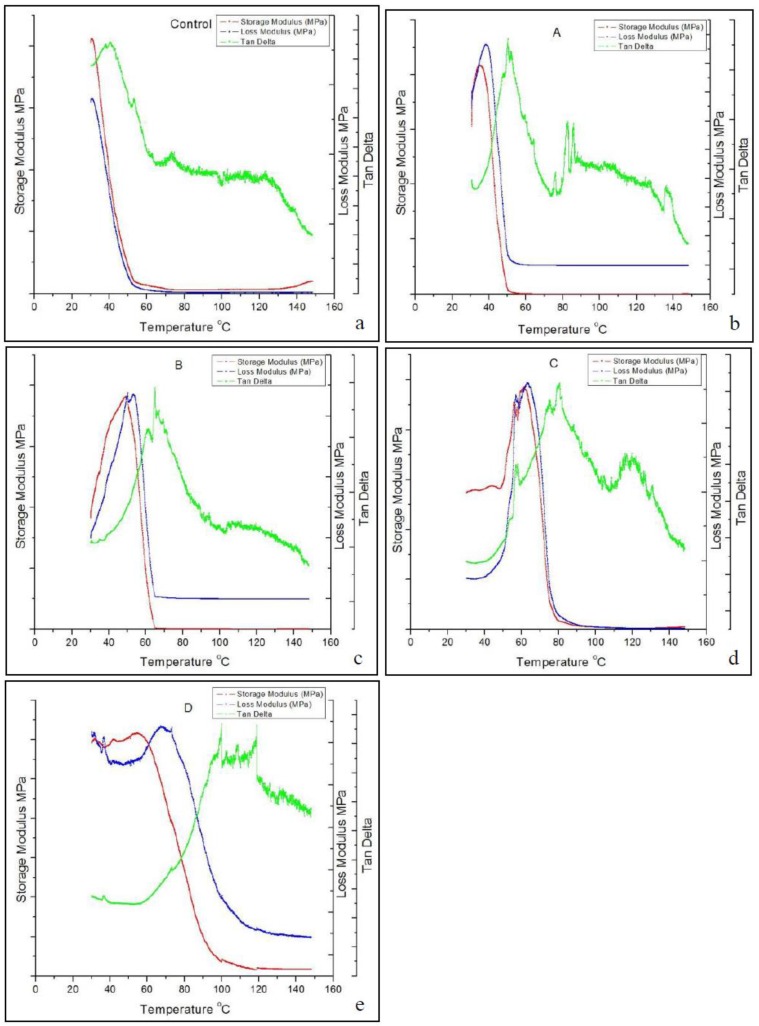
DMA curves of (**a**) PUF control and (**b**–**e**) CNF-PUF with 10–40% CNF replacement ratios.

Although *T*_g_ can be determined using the DSC curve, it was not as sensitive as that measured by the DMA curve [62]. In this study, the *T*_g_ was all determined from the temperature position of the maximum in tan δ in the DMA curve. Figure 6 shows that the *T*_g_ increased from 40 °C for the PUF control up to 100 °C for the CNF-PUF with 40% CNF replacement ratio. The increasing trend of *T*_g_ with the increase of CNF replacement ratio agreed with the results from the DSC curve, except that the *T*_g_ values were slightly different. Possible reasons for the increase of *T*_g_ were that the reaction between CNF and isocyanate resulted in a higher crosslinking density than that between PEG-400 and isocyanate [49]. In addition, as shown in the SEM, the replacement of CNF generated many interlocks between the cells due to the entanglement of long CNF fibers. This more complicated web-like structure limited the mobility of the polyurethane matrix, requiring more energy to reach the glass-to-rubber transition.

### 3.6. Mechanical Properties of PUF and CNF-PUF

**Figure 7:**
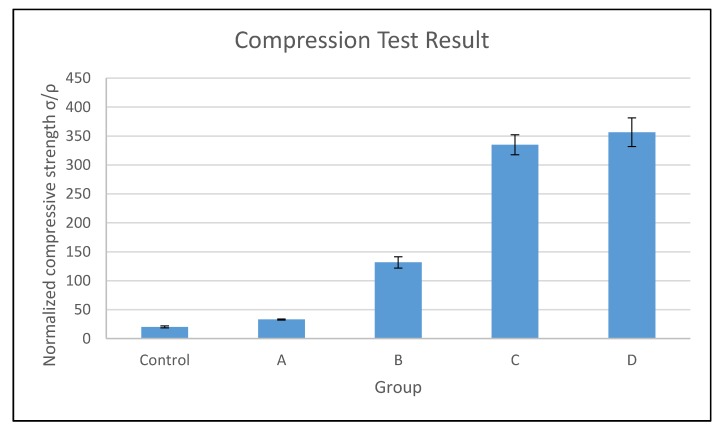
Compression results of PUF control and CNF-PUF with 10–40% CNF replacement ratios.

In this study, the compressive properties of PUF control and CNF-PUF were evaluated. Although the target density (approximately 90 kg/m^3^) was set to be the same during the experiment design, the actual density was slightly varied between different groups of samples. Hence, normalized compressive strength was used to compare the effect of CNF on the mechanical properties. Figure 7 shows that with the increasing CNF replacement ratio, the normalized compressive strength increased up to 18 times that of the PUF control [45]. As discussed in the thermal stability section, the introduction of CNF into the PUF matrix created new urethane bonding between the –OH groups in CNF and the isocyanate, resulting in a higher crosslinking density than in the PUF control [49]. Additionally, the high mechanical properties and web-like entanglement of CNF itself, acting as a filler to the PUF matrix, contributed to the improvement of compressive strength for the CNF-PUF nanocomposites. In this study, a maximum of 40% CNF replacement ratio was reported. Formulations of higher than 40% CNF replacement ratios resulted in a failure of uniform mixing during foam preparation. As shown in Figure 7, the CNF replacement ratio of 30% and 40% did not generate much difference in the normalized compressive strength, indicating that a 30% CNF replacement ratio might be optimal in terms of compressive strength improvement. However, higher amounts of CNF resulted in much greener and more competitive environmentally-friendly foam composites.

## 4. Conclusions

In this study, up to 40% of PEG-400 polyol was replaced with CNF to synthesize PUF. The introduction of CNF disrupted the original open-cell structure of PUF and made the nanocomposite more amorphous. As the CNF replacement ratios increased, large amounts of CNFs deposited on the open-cells and also dispersed among the open-cells, acting as a bridge connecting the cells. The incorporation of CNF also resulted in stronger crosslinking between the CNF-PUF matrix. Evidently, the *T*_g_ increased from 40 °C for the PUF control up to 100 °C for the CNF-PUF with a 40% CNF replacement ratio. Additionally, the introduction of CNF rendered a significant increase in the normalized compressive strength up to 18 times the original value for the PUF control. This study provides an interesting way to synthesize a much greener bio-oriented PUF.

## Figures and Tables

**Table 1:** Foaming formulation.

Chemicals	Parts by weight					Role
	Control	A	B	C	D	
PEG-400	100	90	80	70	60	Polyol
Spray-dried CNF	0	10	20	30	40	Polyol, reinforcing agent
DABCO T12	3	3	3	3	3	Catalyst
DABCO DC5357	1	1	1	1	1	Surfactant
Deionized water	0.8	0.8	0.8	0.8	0.8	Blowing agent
PAPI^TM^ 27	88	89	89	90	90	Reactive prepolymer
